# microRNA‐128‐3p overexpression inhibits breast cancer stem cell characteristics through suppression of Wnt signalling pathway by down‐regulating NEK2

**DOI:** 10.1111/jcmm.15317

**Published:** 2020-06-17

**Authors:** Yuanwen Chen, Nian Wu, Lei Liu, Huaying Dong, Xinao Liu

**Affiliations:** ^1^ Department of General Surgery Chongqing Renji Hospital University of Chinese Academy of Science Chongqing China; ^2^ Department of General Surgery Hainan General Hospital Hainan Medical University Haikou China; ^3^ Clinical laboratory Chongqing Hospital University of Chinese Academy of Science Chongqing China

**Keywords:** breast cancer, cancer stem cells, microRNA‐128‐3p, NIMA‐related kinase 2, self‐renewal, Wnt signalling pathway

## Abstract

Emerging evidence has reported that dysregulation of microRNAs (miRNAs) participated in the development of diverse types of cancers. Our initial microarray‐based analysis identified differentially expressed NEK2 related to breast cancer and predicted the regulatory microRNA‐128‐3p (miR‐128‐3p). Herein, this study aimed to characterize the tumour‐suppressive role of miR‐128‐3p in regulating the biological characteristics of breast cancer stem cells (BCSCs). CD44^＋^CD24^−/low^ cells were selected for subsequent experiments. After verification of the target relationship between miR‐128‐3p and NEK2, the relationship among miR‐128‐3p, NEK2 and BCSCs was further investigated with the involvement of the Wnt signalling pathway. The regulatory effects of miR‐128‐3p on proliferation, migration, invasion and self‐renewal in vitro as well as tumorigenicity in vivo of BCSCs were examined via gain‐ and loss‐of‐function approaches. Highly expressed NEK2 was found in breast cancer based on GSE61304 expression profile. Breast cancer stem cells and breast cancer cells showed a down‐regulation of miR‐128‐3p. Overexpression of miR‐128‐3p was found to inhibit proliferation, migration, invasion, self‐renewal in vitro and tumorigenicity in vivo of BCSCs, which was further validated to be achieved through inhibition of Wnt signalling pathway by down‐regulating NEK2. In summary, this study indicates that miR‐128‐3p inhibits the stem‐like cell features of BCSCs via inhibition of the Wnt signalling pathway by down‐regulating NEK2, which provides a new target for breast cancer treatment.

## 1 INTRODUCTION

Breast cancer is one of the most common cancer types among women in the world with heterogeneity in both morphology and structure.^1^ The main cause of the lethal nature of breast cancer is the malignant invasiveness “metastasis” of the tumour.^2^ Treatment of breast cancer depends on the stage of the disease.^3^ Determination of the treatment of choice is usually contingent upon tumour‐related factors, such as the tumour complication level and patient‐related factors, like the patients' preferences.^4^, ^5^ Mitotic kinesins, whose overexpression has been found in diverse cancers, are demonstrated to serve as markers for the prognosis of breast cancer offering a potential treatment for this disease.^6^ However, treatment of breast cancer still remains a great challenge owing to poor diagnosis and prognosis.^7^ Therefore, it is very important to find new therapeutic targets for breast cancer treatment.

As non‐coding RNAs, microRNAs (miRNAs) regulate the expression of genes at the post‐transcriptional level, including genes that are involved in the development of various types of cancers, including breast cancer.^8^, ^9^ This is clearly exemplified in microRNA‐128‐3p (miR‐128‐3p), which is encoded in miR‐128. miR‐128‐3p is enriched in central nervous system^10^ and has been reported to play an essential role in the prognosis of breast cancer.^11^ As Wnts are associated with proliferation, self‐renewal, and migration of stem cells, the  Wnt signalling pathway is usually regarded as therapeutic targets in different kinds of tumours.^12^ Activating the Wnt signalling pathway can promote the malignant proliferation of breast cancer cells.^13^ NIMA‐related kinase 2 (NEK2) is a kind of mitotic kinases involved in carcinogenesis and development of various cancers. The up‐regulation of NEK2 in several types of tumours indicates that it might serve as a potential target for cancer therapy.^14^ In addition, a previous study has shown that the expression of NEK2 is always up‐regulated in breast cancer.^15^ Although both miR‐128‐3p and NEK2 have been studied in the growth of breast cancer,^16^, ^17^ their exact roles in breast cancer remain unclear. Therefore, this study was designed to investigate the specific relationship between miR‐128‐3p and NEK2 and their effects on the development of breast cancer. In the present study, we hypothesized that overexpression of miR‐128‐3p may regulate the biological characteristics including proliferation, migration and invasion of breast cancer stem cells (BCSCs) by mediating the expression of NEK2.

## 2 MATERIALS AND METHODS

### 2.1 Ethics statement

The study was approved by the Institutional Review Board of Chongqing Renji Hospital, University of Chinese Academy of Science. Written informed consent was obtained from each participant. Nude mice were used for in vivo studies and were cared for in accordance with the *Guide for the Care and Use of Laboratory Animals* published by the National Institutes of Health.

### 2.2 Microarray analysis

The Gene Expression Omnibus (GEO) database (https://www.ncbi.nlm.nih.gov/geo/) was used to search for breast cancer expression profiles, and “limma” package in the R language was used for differential expression analysis with |logFC| > 2 and *P* < 0.05 as screening threshold of differential genes. The heat map of differentially expressed genes was constructed using the “pheatmap” package.

Known breast cancer‐related genes were retrieved in DisGeNET (http://www.disgenet.org/web/DisGeNET/menu), a database with substantial human disease‐related genes and variants that are publicly available. The STRING database (https://string‐db.org/) was employed for correlation analysis of the known genes and differentially expressed genes in breast cancer, and a genetic interaction network was constructed using Cytoscape software.

As a user‐friendly website, UALCAN (http://ualcan.path.uab.edu/index.html) is used in the present study to analyse cancer transcriptome data. In this database, the expression of NEK2 gene in breast cancer was searched. In the DIANA database (http://diana.imis.athena‐innovation.gr/DianaTools/index.php?r=microT_CDS/index), the miRDB database ( http://mirdb.org/miRDB/index.html), the miRSearch database (https://www.exiqon.com/miRSearch) and the TargetScan database (http://www.targetscan.org/vert_71/), the regulatory miRNAs of NEK2 gene were predicted and the results were intersected.

### 2.3 Study participants

Four breast cancer cell lines (mammary carcinoma cell (MCF‐7, ZR‐75‐1, T47D, MB231) and one normal breast epithelial cell line (MCF‐10A) were purchased from Shanghai Institute of Cell Biology, Chinese Academy of Sciences.

A total of 52 cases of married female patients breast cancer who had given birth before (aged 32‐83 years) were admitted in Chongqing Renji Hospital, University of Chinese Academy of Science, where they underwent resection surgery, with cancer tissue and adjacent normal tissues (1.0 cm from the cancer tissues) collected for this study. The painless and hard tumours and breast nodules were found unintentionally or detected in physical examination. None of the patients received anti‐oestrogen treatment. Some tissue specimens were stored at −80°C, and the others were fixed with 10% formalin, dehydrated and embedded in paraffin.

### 2.4 Sorting and identification of BCSCs

Sorting of BCSCs: Human breast cancer cell line MCF‐7 was cultured in an incubator using RPMI‐1640 (Gibco) culture medium containing 10% foetal bovine serum (FBS) at 37°C with 5% CO_2_ and saturated humidity. Collected cells in logarithmic growth phase were made into single‐cell suspension, counted and centrifuged at 503.1 (g) for 10 minutes (*r* = 11 cm) with the supernatant discarded. Then, the cells were resuspended and 1 × 10^7^ cells were mixed with 10 μL CD24‐biotin and incubated at low temperature for 15 minutes. After being rinsed, centrifuged, and resuspended, the cells were mixed and incubated with 20 μL of anti‐biotin MicroBeads at low temperature for 15 minutes. After being rinsed, the cells were centrifuged at   503.1 (g) for 10 minutes (*r* = 11 cm) and resuspended in 500 μL buffer. CD24^−/low^ cells were obtained by negative sorting using magnetic‐activated cell sorting (MACS). CD44^+^CD24^−/low^ cells were obtained through positive sorting from CD24^−/low^ cells using the same method.

Identification of BCSCs: 1. flow cytometry: Cells with good growth status were washed twice with phosphate buffer saline (PBS) and resuspended into single‐cell suspension at a cell concentration of 2 × 10^7^ mL. Then, 50 μL of single‐cell suspension was placed in the flow tube and incubated with the addition of 50 μL of flow buffer containing 5 μL of mouse anti‐human CD24‐fluorescein isothiocyanate (FITC) and 0.625 μL of rabbit anti‐human CD44‐PE at 4°C for 30 minutes with exposure to light avoided and washed twice with flow buffer. The ratio of CD44^+^CD24^−/low^ cells was measured by flow cytometer. After the addition of 5 μL of mouse anti‐human P‐glycoprotein (gp)‐PE, 95 μL of the single‐cell suspension was incubated at 4°C for 30 minutes with exposure to light avoided. The P‐gp level was detected by flow cytometer. The detection results were analysed by EXPO32 ADC Analysis software when the excitation light wavelength was 88 nm. 2. Detection by microsphere culture: Cells with good growth state were resuspended in Dulbecco's modified Eagle's medium (DMEM)/F12 medium containing epidermal growth factor (EGF) (20 ng/mL), basic fibroblast growth factor (bFGF) (10 ng/mL), insulin (5 mg/L) and B27. The cells were seeded into a plastic corning at 2 × 10^4^ mL and cultured at 37°C, 5% CO_2_ and saturated humidity with the culture solution changed once every 2‐3 days. On the 7th day, the number and volume of the microspheres were observed.

### 2.5 Dual‐luciferase reporter assay

The bioinformatics database (microRNA.org; http://www.microrna.org/) was used to predict whether NEK2 is the target gene for miR‐128‐3p. Human embryonic kidney HEK293T cells were cultured in DMEM containing 10% FBS at 5% CO_2_ and 37°C. The complementary DNA (cDNA) fragment of NEK2 3′‐untranslated region (UTR) containing the miR‐128‐3p binding site was inserted into the pmirGLO vector. The cDNA fragment of the binding site‐mutant. NEK2 3′‐UTR was constructed by point mutation and inserted into the pmirGLO vector. The inserted sequence was verified by sequencing. The pmirGLO‐NEK2 or pmirGLO‐mutant (MUT) homeobox C6 (HOXC6) recombinant vector was cotransfected into HEK293T with miR‐128‐3p mimic (miR‐128‐3p overexpression sequence) or miR‐NC (negative control sequence) by lipofection. The cells were incubated for 48 hours, harvested and lysed. The lysate supernatant (100 μL) was taken and added to 100 μL of Renilla luciferase assay solution to detect Renilla luciferase activity. In addition, 100 μL of the lysate supernatant was mixed with 100 μL of firefly luciferase detection reagent to detect the firefly luciferase activity. The SpectraMax M5 multi‐mode plate reader (Molecular Devices) was used to detect Renilla luciferase activity and firefly luciferase activity, respectively with an interval of 2 seconds and a measurement time of 10 seconds.

### 2.6 Cell grouping and transfection

Cells were transfected and grouped into miR‐128‐3p mimic group (transfected with synthetic miR‐128‐3p mimic), miR‐128‐3p mimic‐negative control (NC) group (transfected with mimic NC sequence), miR‐128‐3p inhibitor group (transfected with miR‐128‐3p inhibitor), miR‐128‐3p inhibitor‐NC group (transfected with inhibitor NC sequence), si‐NEK2 group (transfected with siRNA against NEK2), si‐NEK2‐NC group (transfected with NC of siRNA against NEK2), DKK‐1 group (transfected with Wnt pathway inhibitor), dimethyl sulfoxide (DMSO) group (transfected with DKK‐1 solvent control), miR‐128‐3p inhibitor + si‐NEK2 group (transfected with miR‐128‐3p inhibitor and si‐NEK2) and miR‐128‐3p inhibitor + si‐NEK2‐NC group (transfected with miR‐128‐3p inhibitor and NC of siRNA against NEK2). The chemically synthesized miR‐128‐3p mimic, miR‐128‐3p inhibitor and negative control sequences were purchased from Shanghai GenePharma Co., Ltd.. The cells were seeded in a six‐well plate 24 h before transfection. When the cell confluence reached 50%, the human BCSCs were transiently transfected using Lipofectamine 2000 (Invitrogen) and the culture medium was changed after transfection for 6 hours. After 48 hours of culture, the cells were collected for subsequent experiments.

### 2.7 Reverse transcription‐quantitative polymerase chain reaction (RT‐qPCR)

The total RNA was extracted from cells and tissues using an RNA extraction kit (Invitrogen). Designed primers were synthesized by Takara (Table 1). The RNA concentration was measured, and cDNA was synthesized by reverse transcription according to the instructions of the reverse transcription kit (DRR047S, TaKaRa Biotechnology Co. Ltd). The relative expression of miR‐128‐3p was determined with U6 as the internal reference, whereas the other genes were detected with GAPDH as the internal reference.^18^ Each experiment was repeated at least 3 times, and the expression of miR‐128‐3p was calculated by the 2^−ΔΔCt^ method. Using the 2^−△△CT^ method, we calculated the relative transcriptional level of target gene mRNA: △Ct = △CT (experimental group) − △CT (control group); △Ct = Ct (target gene) − Ct (internal reference). Relative transcriptional level of target gene mRNA = 2^−△△CT^.^19^ Each specimen was divided into three parallel tubes and the experiment was conducted three times independently.

**TABLE 1:** Primer sequence for RT‐qPCR

Gene	Primers
miR‐128‐3p	F: 5′‐GGTCACAGTGAACCGGTC‐3′
	R: 5′‐GTGCAGGGTCCGAGGT‐3′
NEK2	F: 5′‐TTATTGACCGGACCAACACC‐3′
	R: 5′‐TCAGGTCCCGATGCAGCACA‐3′
Wnt	F: 5′‐ATCCAGAGAGAAGTCGGCAGCC‐3′
	R: 5′‐CCCGTTGCTCACGAGCGTCTC‐3′
β‐Catenin	F: 5′‐GCTGATACCTTGGCAAAGCA‐3′
	R: 5′‐CAGGATACAGCTCCACAGCA‐3′
CD24	F: 5′‐GGCCAAGCTTATGGGCAGAGCAATGGTG‐3′
	R: 5′‐ATCCCTCGAGTTAAGAGTAGAGATGCAG‐3′
CD44	F: 5′‐AAGGTGGAGCAAACACAACC‐3′
	R: 5′‐GCGAAACCTAATCTCCGACGAA‐3′
Nanog	F: 5′‐GGATGTCTTTAGATCAGAGGATGCCC‐3′
	R: 5′‐CCACAGAAAGAGCAAGACACCAACC‐3′
Oct‐4	F: 5′‐CGTGAAGCTGGAGAAGGAGAAGCTG‐3′
	R: 5′‐CCACATCGGCCTGTGTATATCCCAG‐3′
U6	F: 5′‐CTCGCTTCGGCAGCACATA‐3′
	R: 5′‐GTGCAGGGTCCGAGCT‐3′
GAPDH	F: 5′‐AGGTCGGAGTCAACGGATTTG‐3′
	R: 5′‐GTGATGGCATGGACTGTGGT‐3′

Abbreviations: F, forward; R, reverse; NEK2, NIMA‐related kinase 2; Wnt, Wingless and INT‐1; CD24, cluster of differentiation 24; CD44, cluster of differentiation 44; Oct‐4, octamer‐binding transcription factor 4; GAPDH, glyceraldehyde‐3‐phosphate dehydrogenase.

### 2.8 Western blot analysis

The total protein of tissues and cells was extracted. After ice bathing and centrifugation, the sample was added to each lane for electrophoresis separation. The separated protein was transferred onto a nitrocellulose membrane. Then, the nitrocellulose membrane was blocked with 5% skim milk powder at 4°C overnight and incubated overnight with primary rabbit anti‐mouse polyclonal antibodies diluted at the ratio of 1:1000: NEK2 (ab115731), β‐catenin (ab16051), Nanog (ab21624), Oct‐4 (ab19857), CXCL16 (ab101404), EGFR (ab52894), TCF‐4 (ab217668) and Pygo2 (ab109001) and rabbit anti‐mouse monoclonal antibodies diluted at 1:1000: CD24 (ab64064), and CD44 (ab51037), all of which were purchased from Abcam Inc, Cambridge, UK. The membrane was washed 3 times with PBS at room temperature, 5 minutes for each, incubated with oscillation with the addition of the secondary antibody of horseradish peroxidase (HRP)‐labelled goat anti‐rabbit immunoglobulin (IgG) (1:10 000, ab6728, Boster Biological Technology Co. Ltd, Wuhan, China) at 37°C for 1 hour, washed with Tris‐buffered saline‐Tween (TBST) and visualized using HRP electroluminescence (ECL). Grey value analysis of the target bands was performed using ImageJ software, and the experiment was repeated three times independently.

### 2.9 TOP/FOP Flash

Breast cancer stem cells were seeded in the 96‐well plates, and 24 hours later, transfection plasmids, TOPFlash or FOPFlash, and internal reference pRL‐TK plasmids (Promega) were added to each well according to the instructions of Lipofectamine 2000 (11668019, Thermo Fisher Scientific). The plasmids were gently mixed with 100 μL of L‐DMEM and placed at room temperature for 5 minutes. Then, 0.5 μL of Lipofectamine 2000 was gently mixed with 100 μL of L‐DMEM and placed for 5 minutes at room temperature. After the original medium was removed, the cells were washed with L‐DMEM and added to the mixed transfection solution. And after 6 hours of transfection, the cells were cultured in complete medium. The culture medium was discarded after 24‐48 hours, and the firefly fluorescence activity and the Renilla fluorescence activity in each well were detected using a Dual‐Luciferase^®^ Reporter Assay System (E1910, Promega). The ratio of firefly fluorescence activity to the Renilla fluorescence activity indicates the activation level of transcription factors in the Wnt/β‐catenin signalling pathway in the cell.^20^

### 2.10 Immunofluorescence staining

After conventional treatment, transfection, and counting, the cells were cultured in an immunofluorescence chamber with 2 × 10^5^ cells per well. When cell confluence rate reached about 90%, the cells were washed 3 times with PBS (this process was performed on ice), fixed with 4% paraformaldehyde (1 mL per well), and placed at room temperature for 15 minutes. After being washed with PBS for 3 times, the cells were perforated with 0.3% Triton. After 10 minutes, the cells were washed 3 times with PBS, blocked with goat serum, and placed for 30 minutes. After incubation with the primary antibody prepared with PBS at 4°C overnight, the cells were washed 3 times with PBS, incubated with the secondary antibody at room temperature for 1 hour avoiding exposure to light, washed 3 times with PBS, stained with 4′,6‐diamidino‐2‐phenylindole (DAPI) avoiding exposure to light for 15 minutes. After being washed 3 times with PBS in the dark, the cells were mounted with fluorescent quencher and photographs were taken under a fluorescence microscope.

### 2.11 **5‐Ethynyl‐2**′**‐deoxyuridine (EdU) assay**

Logarithmic growth‐phase cells were seeded in the 96‐well plates with 4 × 10^3^ ~ 1 × 10^5^ cells per well and cultured to normal growth stage. Each well was incubated with 100 μL of 50 μM EdU medium for 2 hours. Cells in each well were incubated with 50 μL of cell fixative (PBS containing 4% paraformaldehyde) for 30 minutes at room temperature and incubated with 50 μL of 2 mg/mL glycine for 5 minutes. After that, 100 μL of 1 × Apollo^®^ staining reaction solution was added to each well and then incubated in the dark at room temperature for 30 minutes. Next, 100 μL of penetrant (PBS containing 0.5% Triton X‐100) was used for washing for 2‐3 times, each for 10 minutes. The plate was then incubated with 100 μL of 1 × Hoechst 33 342 reaction solution for 30 minutes. Under a fluorescence microscope, the number of EDU‐positive staining cells was observed to calculate cell proliferation. The experiment was repeated for three times.

### 2.12 Transwell assay

Cell migration detection: When cell confluence reached about 80%, the third‐generation cells were starved with serum‐free DMEM for 24 hours. The cells were placed in the lower chamber of the Transwell chamber (Corning Glass Works) with serum‐free DMEM at 37°C for 1 hour. After cell attachment, cells were resuspended in serum‐free DMEM, counted and diluted to 3 × 10^5^/mL. The cells (100 μL) were transferred to the Transwell upper chamber, and 600 μL of DMEM containing 10% serum was added to the lower chamber. According to Transwell chamber instructions, the cells were incubated for 24 hours. After that, the cells in the lower chamber were stained with 0.1% crystal violet solution for 10 minutes and photographed under the inverted microscope (Olympus Optical Co., Ltd) with six visual fields selected, followed by cell counting. The experiment was repeated three times. Cell invasion was detected as follows: The Matrigel preserved at −20°C was taken out and melt at 4°C. Afterwards, the Matrigel was diluted with serum‐free DMEM at a ratio of 1:10 with cell concentration adjusted to 1.0 × 10^5^ cells/mL. The rest procedures were the same as cell migration detection. The experiment was repeated three times.

### 2.13 Sphere formation assay

A total of 1 × 10^4^ BCSCs were seeded into the low‐adsorption 96‐well plates and cultured in serum‐free DMEM/F12 medium containing 20 ng/mL EGF and 20 ng/mL FGF‐β with the solution semi‐quantitatively changed every 2 days. After continuous culture for 10 days, the BCSCs were photographed under the inverted microscope and counted.

### 2.14 Soft agar colony formation assay

Agarose at 0.7% low melting point was prepared in fresh DMEM and stored at 4°C for subsequent use. The 0.7% agarose was heated and melted, and 2 mL agarose was transferred to a petri dish with the diameter of 100 mm, and gently shaken until the agarose covered the bottom of the dish evenly, which was then cooled and solidified for use. Cell inoculation, culture and colony identification: 1 mL of cell suspension and an equal volume of 0.7% agarose solution were diluted to 0.35% agarose cell mixture, and about 1 × 10^4^ cells were seeded into each 100‐cm^2^ mixture. Three parallels were set in each group. After solidification of the upper layer of agar, 2‐3 mL of the culture solution was added on the surface of the agar softly to avoid crushing. The cells were cultured at 37°C and 5% CO_2_ with the solution changed once every 2‐3 days, and the culture was terminated after 1 month. The culture dish was photographed under an inverted microscope for cell counting, and only the cluster with ≥50 cells was determined as a cell colony.

### 2.15 Limiting dilution assay (LDA) in vivo and subcutaneous tumour formation in NOD‐SCID mice

The cells were seeded in a low‐adherence culture plate, and after 7 days of culture, BCSC spheres in each group were collected, centrifuged in a 10‐mL glass centrifuge tube with the removal of the supernatant, and washed once with saline. The BCSC spheres were added to 1 mL of 0.5% trypsin, incubated at 37°C with the incubator bottom flicked every 2 minutes, treated for about 10 minutes, and decomposed into single cells. After termination of attachment with the addition of 3 mL complete medium, the single cells were centrifuged to collect cell precipitation, washed once with saline, added to a suitable amount of normal saline and gently pipetted into single‐cell suspension, followed by cell counting. Different amounts of cells (1 × 10^3^, 5 × 10^3^, 1 × 10^4^, or 5 × 10^4^) were resuspended in 50 μL normal saline, mixed with 50 μL Matrigel Matrix (1:1), and inoculated subcutaneously into non‐obese diabetic (NOD)‐severe combined immunodeficiency (SCID) mice. Two weeks after the inoculation, tumour formation was observed and recorded. The proportion of tumour stem cells was calculated using extreme limiting dilution analysis (ELDA) software (http://bioinf.wehi.edu.au/software/elda/index.html).^21^ Subcutaneous tumour formation in nude mice was evaluated. The cells (2 × 10^6^) were resuspended in 50 μL of normal saline, then mixed with 50 μL Matrigel Matrix (1:1) and inoculated subcutaneously into NOD‐SCID mice. The volume of the tumour was observed and recorded 1 week after the inoculation, and the tumour growth was recorded weekly.

### 2.16 Statistical analysis

Statistical analysis was performed using the SPSS 21.0 software (IBM Corp.). Normal distribution and homogeneity of variance tests were conducted concerning all data. The enumeration data were expressed using cases or percentage (%), and the measurement data were presented as the mean ± standard deviation. Comparisons between two groups were analysed using *t* test, and Welch's correction was used for unequal variances. Data analysis among multiple groups was performed by one‐way analysis of variance. The data analyses at different time‐points were performed using repeated‐measures analysis of variance. The data of skewed distribution were analysed by rank‐sum test. All experiments were repeated three times. A *P* < 0.05 was considered statistically significant.

## 3 RESULTS

### 3.1 NEK2 and miR‐128‐3p are involved in the development of breast cancer

**FIGURE 1:**
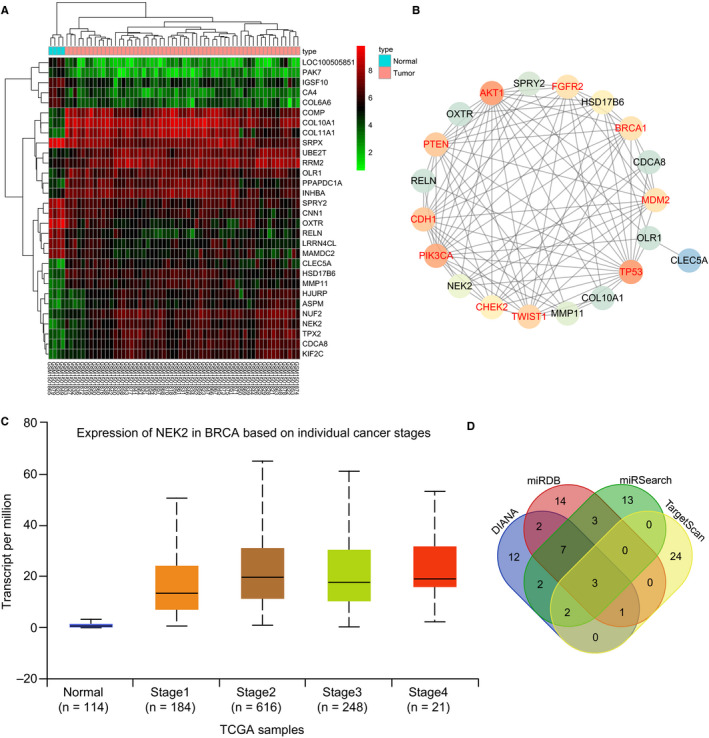
miR‐128‐3p was predicted to regulate the development of breast cancer via the mediation of NEK2. A, Heat map of the top 30 genes with significantly differential expression in expression profile GSE61304, in which the abscissa indicates the sample number, the ordinate indicates the gene name, the upper dendrogram indicates the sample type cluster, and the left dendrogram indicates the clustering of gene expression levels. Each small square in the figure indicates the expression level of a gene in one sample, and the right upper histogram is the colour scale. B, Correlation analysis of the top 10 significantly differential genes and 10 known genes of breast cancer expression profile. Each small circle in the figure represents a gene, and the line between the circles indicates the interaction between the two genes. The red font indicates the known genes for breast cancer obtained in the database, and the black font indicates differentially expressed genes for breast cancer. The brighter the circle, the higher the core level of the gene in the network map. C, Expression of NEK2 gene in the breast cancer database included in TCGA. The abscissa indicates the sample type, the ordinate indicates the gene expression, the first box plot refers to the normal sample, and the next four box plots indicate the expression of the NEK2 gene in the breast cancer in different periods. D, Prediction of regulatory miRNA of NEK2 gene. The four ellipses in the figure represent the prediction of miRNA regulating NEK2 in four databases, and the middle part represents the intersection of four databases

From the GEO database, the breast cancer expression profile GSE61304 was retrieved. Through differential expression analysis of the breast cancer samples and normal control samples in the expression profile, 177 differentially expressed genes were obtained, of which 58 differential genes were highly expressed, whereas 119 differential genes were down‐regulated in breast cancer. The heat map of expression of the first 30 differentially expressed genes was constructed (Figure 1A). To further screen for breast cancer‐associated genes, the known genes for breast cancer were searched in the DisGeNET database and the top 10 genes with the highest scores were selected for subsequent analysis (Table 2). Correlation analysis of the first 10 significant differential genes in the GSE61304 expression profile and the known genes of breast cancer was conducted and the gene interaction network map was constructed (Figure 1B), finding that among the top 10 differential genes, NEK2 gene was at the core of the network map and had an interaction with genes known to be related to breast cancer such as AKT1 and BRCA1. The expression level of NEK2 gene was further analysed in breast cancer samples included by The Cancer Genome Atlas (TCGA) (Figure 1C), showing that the expression level of NEK2 gene was significantly up‐regulated in the breast cancer samples at different stages compared with the control group. In addition, NEK2 gene has been reported to promote tumour development through the Wnt signalling pathway.^22^ In order to further understand the upstream regulation mechanism of NEK2 gene, the regulatory miRNAs of NEK2 were predicted through DIANA, miRDB and other databases (Figure 1D). Three miRNAs mutually emerged from the four databases, among which miR‐128‐3p has the highest scoring. In summary, in breast cancer, miR‐128‐3p is likely to influence the development of breast cancer via the Wnt signalling pathway by regulating the NEK2 gene.

**TABLE 2:** Known genes of breast cancer

Gene	Gene name	Score	PMIDs
PIK3CA	Phosphatidylinositol‐4,5‐bisphosphate 3‐kinase catalytic subunit alpha	0.28	301
BRCA1	BRCA1, DNA repair associated	0.28	2228
TP53	Tumour protein p53	0.28	1099
PTEN	Phosphatase and tensin homolog	0.248	173
AKT1	AKT serine/threonine kinase 1	0.242	152
CHEK2	Checkpoint kinase 2	0.236	130
FGFR2	Fibroblast growth factor receptor 2	0.231	112
MDM2	MDM2 proto‐oncogene	0.224	86
CDH1	Cadherin 1	0.222	71
TWIST1	Twist family bHLH transcription factor 1	0.213	47

Note

The score of the reliability of the gene‐disease pair is based on the reported type and number of sources and PMIDs; PMIDs refer to the total number of PMIDs supporting the association.

### 3.2 CD44^+^CD24^−/low^ exhibits high self‐renewal capacity

**FIGURE 2:**
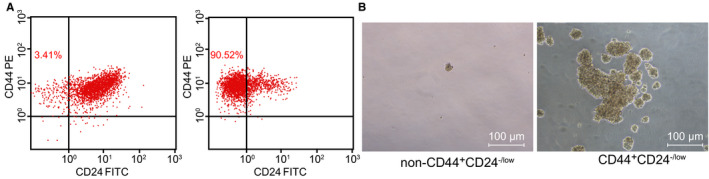
CD44^+^CD24^−/low^ cells were selected for the experiments, as CD44^+^CD24^−/low^ cells were consistent with the high self‐renewal capacity of BCSCs. A, Flow cytometric analysis of the proportion of CD44^+^CD24^−/low^ cells in MCF‐7 cell line (left panel) and purification of CD44^+^CD24^−/low^ cells from CD44^+^CD24^−/low^ cell subsets sorted by flow cytometry (right panel); B, Sphere‐forming ability of different cell subsets (non‐CD44^+^CD24^−/low^ cell subsets (as control) and CD44^+^CD24^−/low^ cells) in MCF‐7 cell line (×100)

The number of cells was counted before and after cell sorting by MACS method. The flow cytometric data showed that the ratio of CD44 + CD24^−/low^ cells in MCF‐7 cells was 3.41%. After purification of CD44^+^CD24^−/low^ cells in the sorted CD44^+^CD24^−/low^ cell subset, we obtained a CD44^+^CD24^−/low^ cell ratio of over 90% (Figure 2A), which was qualified for the requirements of further experiments. To further identify the sorted cells, the sorted CD44^+^CD24^−/low^ cells and non‐CD44^+^CD24^−/low^ cells were cultured in serum‐free DMEM/F12 medium containing EGF, bFGF, B27 and insulin, respectively. After 7 days, the non‐CD44^+^CD24^−/low^ cell subset had fewer formed microspheres, and the formed microspheres were smaller in volume. The CD44^+^CD24^−/low^ cell subset grew in a suspended state with more microspheres formed, and the microspheres were larger (Figure 2B). These observations demonstrated that the CD44^+^CD24^−/low^ group cells were consistent with the high self‐renewal ability of BCSCs, indicating that we successfully isolated BCSCs.

### 3.3 miR‐128‐3p is poorly expressed in BCSCs and breast cancer cells

**FIGURE 3:**
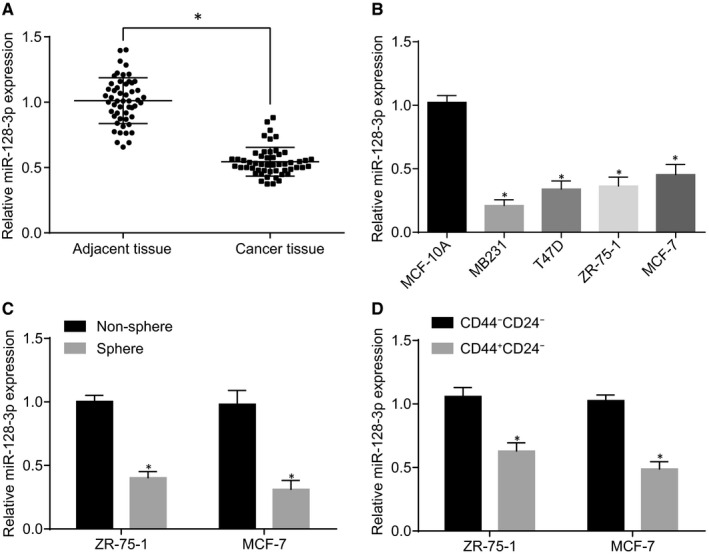
miR‐128‐3p is down‐regulated in BCSCs and breast cancer cells. A, Relative expression of miR‐128‐3p in breast cancer tissues and adjacent tissues (*P < 0.05 compared with adjacent tissue); B, Relative expression of miR‐128‐3p in breast cell lines (*P < 0.05 compared with MCF‐10A); C, Relative expression of miR‐128‐3p in non‐spheroids and spheroids (*P < 0.05 compared with non‐spheroids); D, Relative expression of miR‐128‐3p in CD44^−^CD24^−^ and CD44^+^CD24^−^ (*P < 0.05 compared with CD44^−^CD24^−^); U6 was used as the internal reference of the expression level of miR‐128‐3p and GAPDH was as the internal reference of NEK2, Wnt, β‐catenin, CD24, CD44, Nanog, Oct‐4 and Pygo2. Themeasurement data are expressed in the form of mean ± standard deviation. Difference analysis between two groups was performed by *t* test, and the data analysis among multiple groups was performed by one‐way analysis of variance

The present study selected 52 breast cancer patients to detect the expression of miR‐128‐3p in breast cancer tissues and adjacent tissues using RT‐qPCR, whose results showed that the expression of miR‐128‐3p was lower in breast cancer tissues than in adjacent tissues (Figure 3A). The expression of miR‐128‐3p in several breast cancer cells was also detected, showing that the expression of miR‐128‐3p in MCF‐7, ZR‐75‐1, T47D and MB231 was down‐regulated in comparison with that in MCF‐10A (Figure 3B). To further verify the expression of miR‐128‐3p in BCSCs, the breast cancer cells were cultured in low‐adhesion plates to enrich BCSCs. The expression of miR‐128‐3p was detected and the results showed that miR‐128‐3p expression was reduced in the BCSC sphere in comparison with the non‐sphere (Figure 3C). CD44 and CD24 are common markers of BCSCs; therefore, positive and negative cells of CD44 and CD24 were sorted from MCF‐7 and ZR‐75‐1 cells by flow cytometry, and miR‐128‐3p expression was determined. Sorting results showed that miR‐128‐3p has lower expression in the CD44^+^CD24^−^ cell subset compared to CD44^−^CD24^−^ subset (Figure 3D).

### 3.4 Overexpressed miR‐128‐3p inhibits proliferation, migration and invasion of BCSCs

**FIGURE 4:**
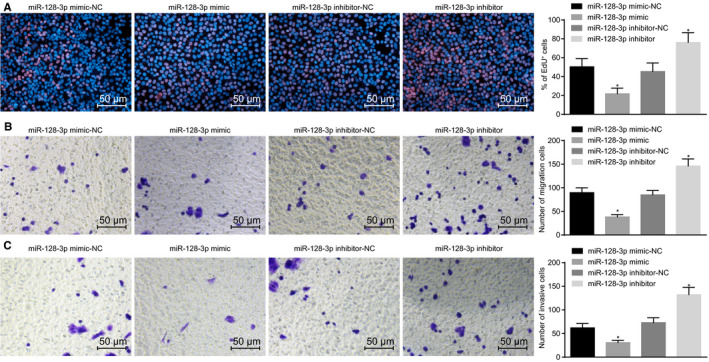
Overexpressing miR‐128‐3p decreases proliferation, migration and invasion of BCSCs. A, Cell proliferation ability after transfection detected by EdU assay (×200); B, Cell migration ability detected by Transwell assay (×200); C, cell invasion ability detected by Transwell assay (×200); *P < 0.05 compared with the corresponding NC group; the experiment was repeated three times, the measurement data are expressed in the form of mean ± standard deviation, and the data analysis among multiple groups was performed by one‐way analysis of variance

EdU assay was applied to analyse the effect of miR‐128‐3p on the proliferation of BCSCs and the results (Figure 4A) showed that after inhibition of miR‐128‐3p, the proportion of EdU‐positive cells was significantly higher than that in response to inhibition of miR‐128‐3p‐NC. Whereas with overexpression of miR‐128‐3p, the positive cells have significantly decreased, suggesting that overexpression of miR‐128‐3p inhibits the synthesis of nascent DNA, hence inhibiting cell proliferation. The results of the migration and invasion of cells detected by Transwell showed that with overexpression of miR‐128‐3p, the migration and invasion of cells have significantly decreased compared to the miR‐128‐3p‐mimic‐NC group (*P* < 0.05). Moreover, in comparison with the miR‐128‐3p‐inhibitor‐NC group, the miR‐128‐3p inhibitor group exhibited promoted cell migration and invasion (*P* < 0.05) (Figure 4B,C). The results indicate that overexpression of miR‐128‐3p suppressed proliferation, migration and invasion of BCSCs.

### 3.5 Up‐regulation of miR‐128‐3p inhibits self‐renewal and amplification of BCSCs

**FIGURE 5:**
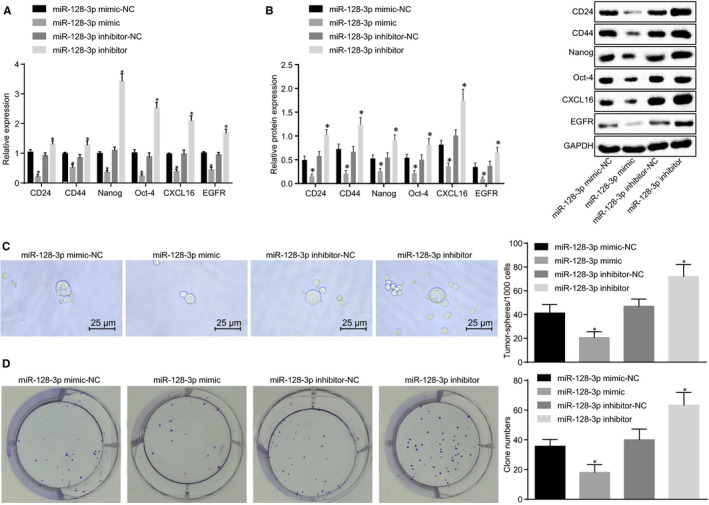
Overexpression of miR‐128‐3p reduces self‐renewal and amplification of BCSCs. A, mRNA expression of BCSC‐related markers after transfection detected by RT‐qPCR; B, Protein expression of BCSC‐related markers after transfection detected by Western blot analysis; C, Sphere‐forming ability of BCSCs after transfection detected by cell sphere formation (×400); D, Colony‐forming ability of BCSCs after transfection detected by colony formation assay; *P < 0.05 compared with the corresponding NC group; the experiment was repeated three times, the measurement data are expressed in the form of mean ± standard deviation, and the data analysis among multiple groups was performed by one‐way analysis of variance

RT‐qPCR and Western blot analyses were performed to determine the mRNA and protein expression of surface markers (CD24 and CD44) and stemness transcription factors (Nanog and Oct‐4) of BCSCs after transfection. The results showed that compared with the corresponding NC groups, overexpressed miR‐128‐3p exhibited decreased CD24, CD44, Nanog and Oct‐4, which was opposite to the trend of inhibited miR‐128‐3p (Figure 5A,B). Through cell sphere formation analysis, which was carried out to detect the sphere‐forming ability of BCSCs, the miR‐128‐3p inhibitor group was found to have increased number of spheroids compared to the corresponding NC group, whereas the miR‐128‐3p mimic group exhibited a decreased number of spheroids, indicating that overexpression of miR‐128‐3p inhibited self‐renewal of BCSCs (Figure 5C). In addition, we also performed soft agar colony formation to detect the colony‐forming ability of BCSCs. The results showed that the number of clones formed by cells has increased when miR‐128‐3p was inhibited, and the number of clones formed by cells has significantly decreased when miR‐128‐3p was overexpressed, revealing that overexpression of miR‐128‐3p inhibited the amplification of BCSCs (Figure 5D). The aforementioned results provide evidence that self‐renewal of BCSCs could be suppressed in response to the up‐regulation of miR‐128‐3p.

### 3.6 Overexpression of miR‐128‐3p inhibits tumorigenicity and tumour growth of BCSCs in vivo

**FIGURE 6:**
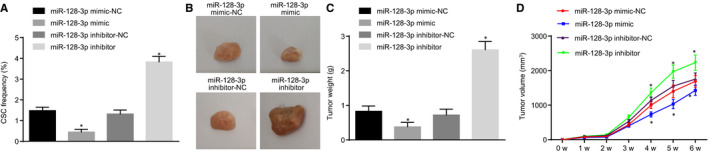
Overexpression of miR‐128‐3p inhibits tumorigenicity and tumour growth of BCSCs in vivo. A, Effects of miR‐128‐3p on the proportion of stem cells in BCSCs in vivo detected by LDA in vivo; B, Size of tumour cells in nude mice after transfection; C, tumour weight in nude mice; D, Volume change of tumour formation in nude mice; *P < 0.05 compared with the corresponding NC group; the experiment was repeated three times. The measurement data are expressed in the form of mean ± standard deviation, and the data analysis among multiple groups was performed by one‐way analysis of variance. The data analysis at different time‐points was performed using repeated‐measures analysis of variance

To further validate the regulation of miR‐128‐3p on BCSCs, we investigated the effects of miR‐128‐3p in vivo. Initially, LDA in vivo was applied to examine the effect of miR‐128‐3p on tumorigenicity and stem cell ratio in BCSCs. The results showed that the number of tumours and cancer stem cell proportion was significantly higher when miR‐128‐3p was inhibited than that of the corresponding NC group (Table 3, Figure 6A). In addition, we further explored the effect of miR‐128‐3p on tumour growth using a mouse subcutaneous tumour model. After BCSC enrichment in low‐adhesion cell cultures, the stem cell spheroids were collected, discomposed into single cells, and inoculated subcutaneously into NOD‐SCID mice at 2 × 10^6^ cells/dot. The tumour growth was observed continuously with the tumour volume recorded. The results showed that compared with the miR‐128‐3p inhibitor‐NC group, in the miR‐128‐3p inhibitor, the tumour formation time was earlier with faster tumour growth rate and larger tumours. In comparison with the miR‐128‐3p mimic‐NC group, the miR‐128‐3p mimic group showed late tumour formation time with a slower growth rate and smaller tumours (Figure 6B‐D) (*P* < 0.5).

**TABLE 3:** Tumorigenicity of BCSC spheroids

Injected cells	miR‐128‐3p mimic‐NC	miR‐128‐3p mimic	miR‐128‐3p inhibitor‐NC	miR‐128‐3p inhibitor
1000	0/3	0/3	0/3	2/3
5000	1/3	0/3	1/3	2/3
10 000	2/3	1/3	2/3	3/3
50 000	2/3	2/3	2/3	3/3
Total	5/12	3/12	5/12	10/12

Abbreviations: NC, negative control; miR, microRNA.

### 3.7 NEK2 is a direct target gene of miR‐128‐3p

**FIGURE 7:**
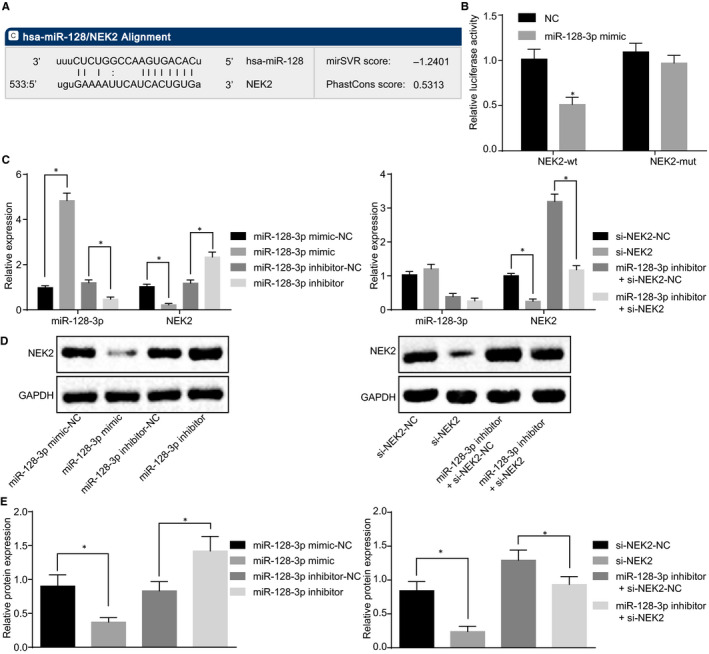
NEK2 is a direct target gene of miR‐128‐3p. A, The targeting relationship between miR‐128‐3p and NEK2 predicted in microRNA.org; B, The targeting relationship between miR‐128‐3p and NEK2 verified by dual‐luciferase reporter assay; C, Relative expression of miR‐128‐3p and NEK2 in each group detected by RT‐qPCR; D, Grey value analysis of NEK2; E, protein expression of NEK2 detected by Western blot analysis; *P < 0.5 compared with the NC group; the measurement data are expressed as mean ± standard deviation, difference analysis between two groups was performed by *t* test, and the data analysis among multiple groups was performed by one‐way analysis of variance. The experiment was repeated three times

According to an analysis by online analysis software, a specific binding region was identified between the 3′‐UTR of the NEK2 gene and the miR‐128‐3p sequence (Figure 7A). The target relationship between NEK2 and miR‐128‐3p was verified using dual‐luciferase reporter assay (Figure 7B). The results showed that overexpression of miR‐128‐3p has significantly inhibited the luciferase activity of 3′‐UTR in NEK2 wild‐type (WT) compared to the NC group (*P* < 0.05), whereas miR‐128‐3p had no significant effect on the luciferase activity of 3′‐UTR in the NEK2‐MUT (*P* > 0.05). The results of RT‐qPCR (Figure 7C) and Western blot analysis (Figure 7D,E) showed that compared to the corresponding NC group, the mRNA and protein expression of NEK2 has significantly decreased after overexpressing miR‐128‐3p (all *P* < 0.05), whereas there was no significant difference in the expression of miR‐128‐3p in the si‐NEK2 group (*P* > 0.05). Furthermore, compared to the corresponding NC group, mRNA and protein expression of NEK2 has significantly decreased in the si‐NEK2 group (*P* < 0.05); when miR‐128‐3p was inhibited, the mRNA and protein expression of NEK2 was significantly increased (*P* < 0.05). Compared with the miR‐128‐3p inhibitor + si‐NEK2‐NC group, the expression of miR‐128‐3p was not significantly changed in the miR‐128‐3p inhibitor + si‐NEK2 group (*P* > 0.05), but the mRNA and protein expression of NEK2 was significantly decreased (both *P* < 0.05). These results prove that miR‐128‐3p specifically binds to NEK2 3′‐UTR and down‐regulates NEK2 gene expression at the post‐transcriptional level.

### 3.8 miR‐128‐3p inhibits proliferation, migration and invasion of BCSCs by silencing NEK2

**FIGURE 8:**
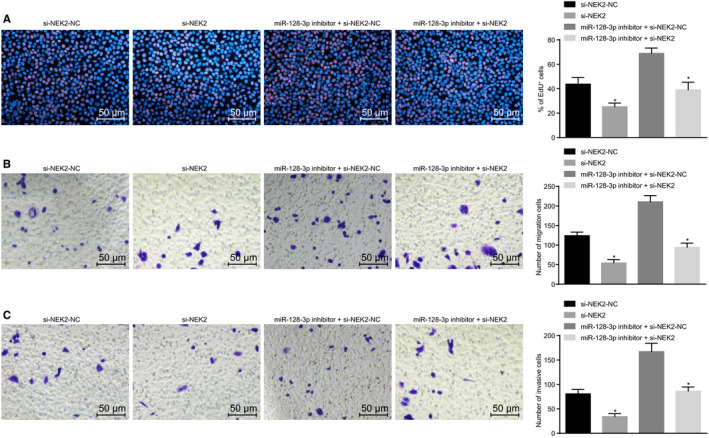
miR‐128‐3p reduces proliferation, migration and invasion of BCSCs by down‐regulating NEK2. A, Cell proliferation ability after transfection detected by EdU assay (×200); B, Cell migration ability detected by Transwell assay (×200); C, Cell invasion ability detected by Transwell assay (×200); *P < 0.05 compared with the corresponding NC group; the measurement data are expressed in the form of mean ± standard deviation, and the data analysis among multiple groups was performed by one‐way analysis of variance. The experiment was repeated three times

The results of the EdU assay (Figure 8A) showed that the proportion of EdU‐positive cells in the si‐NEK2 group was significantly lower than that in the corresponding NC group; compared with the miR‐128‐3p inhibitor + si‐NEK2‐NC group, the proportion of positive cells in the miR‐128‐3p inhibitor + si‐NEK2 group was also decreased significantly, indicating that silencing NEK2 can inhibit the synthesis of nascent DNA, thus repressing cell proliferation. Transwell results which determined the migration and invasion of cells showed (Figure 8B,C) that in the si‐NEK2 group, the migration and invasion were significantly reduced compared with the corresponding NC group (*P* < 0.05); compared with the miR‐128‐3p inhibitor + si‐NEK2‐NC group, the cell migration and invasion ability of the miR‐128‐3p inhibitor + si‐NEK2 group were also significantly decreased (*P* > 0.05). The results indicate that silencing NEK2 can attenuate the migration and invasion ability of BCSCs. All these results indicate that miR‐128‐3p can decrease proliferation, migration and invasion of BCSCs by down‐regulating NEK2.

### 3.9 miR‐128‐3p inhibits self‐renewal of BCSCs by down‐regulating NEK2

**FIGURE 9:**
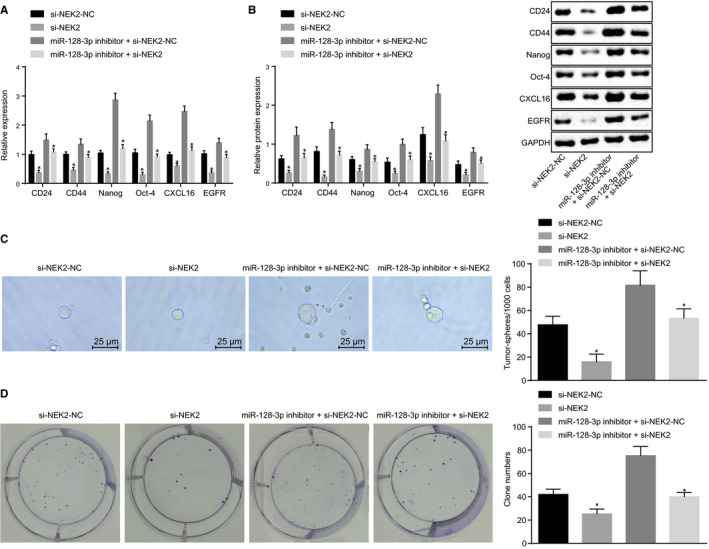
miR‐128‐3p decreases self‐renewal of BCSCs by down‐regulating NEK2. A, mRNA expression of BCSC‐related markers after transfection detected by RT‐qPCR; B, Protein expression of BCSC‐related marker after transfection detected by Western blot analysis; C, Sphere‐forming ability of BCSCs after transfection by sphere formation assay (×400); D, the colony‐forming ability of BCSCs after transfection detected by colony formation assay; *P < 0.05 compared with the corresponding NC group; the measurement data are expressed as mean ± standard deviation, and the data analysis among multiple groups was performed by one‐way analysis of variance. The experiment was repeated three times

RT‐qPCR and Western blot analyses were conducted for the determination of mRNA and protein expression of surface markers (CD24 and CD44) and stemness transcription factors (Nanog and  Oct‐4) of BCSCs after transfection. In addition, sphere formation and soft agar colony formation were conducted to detect the ability of sphere formation and colony formation respectively. The results showed that the mRNA and protein expression of CD24, CD44, Nanog and Oct‐4 was decreased in the si‐NEK2 group compared to the corresponding NC group (Figure 9A,B), and sphere formation, colony formation and proliferative capacity were decreased significantly (Figure 9C,D). Compared with the miR‐128‐3p inhibitor + si‐NEK2‐NC group, the mRNA and protein expression of CD24, CD44, Nanog and Oct‐4 (Figure 9 A,B), and the sphere‐forming ability, colony‐forming ability and cell proliferation (Figure 9C,D) were also declined in the miR‐128‐3p inhibitor + si‐NEK2 group. These results suggest that miR‐128‐3p inhibits self‐renewal of BCSCs by down‐regulating NEK2.

### 3.10 miR‐128‐3p inhibits tumorigenicity and tumour growth of BCSCs by down‐regulating NEK2

**FIGURE 10:**
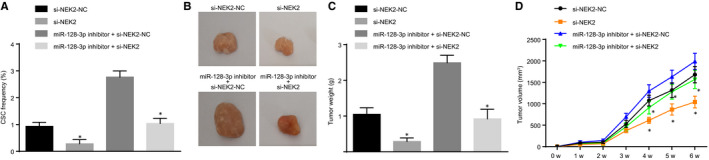
miR‐128‐3p represses tumorigenicity and tumour growth of BCSCs via silencing of NEK2. A, The ratio of stem cells in mice measured by LDA in vivo; B, The size of tumour cells in nude mice after transfection; C, tumour weight in nude mice; D, Volume of tumour formation in nude mice; *P < 0.05 compared with the corresponding NC group; the measurement data are expressed in the form of mean ± standard deviation, and the data analysis among multiple groups was performed by one‐way analysis of variance. The data analysis at different time‐points was performed using repeated‐measures analysis of variance. The experiment was repeated three times

Limiting dilution assay in vivo was used to detect the number of tumours and the proportion of tumour stem cells. The results showed that the number of tumours and the proportion of tumour stem cells were lower in the si‐NEK2 group compared with those in the corresponding NC group; compared with those in the miR‐128‐3p inhibitor + si‐NEK2‐NC group, the number of tumours and the proportion of tumour stem cells were low in the miR‐128‐3p inhibitor + si‐NEK2 group (Table 4, Figure 10A). The subcutaneous tumour‐bearing model of mice showed that compared with the blank group and the NC group, the si‐NEK2 group exhibited shorter tumour formation time with the slow growth rate and smaller tumours. Compared with the miR‐128‐3p inhibitor + si‐NEK2‐NC group, the miR‐128‐3p inhibitor + si‐NEK2 group had a shorter tumour formation time, slower growth rate, and smaller tumours (Figure 10B‐D). These results revealed that up‐regulated miR‐128‐3p could repress tumorigenicity and tumour growth of BCSCs by down‐regulating NEK2.

**TABLE 4:** Tumorigenicity of BCSC spheroids

Injected cells	si‐NEK2‐NC	si‐NEK2	miR‐128‐3p inhibitor + si‐NEK2‐NC	miR‐128‐3p inhibitor + si‐NEK2
1000	0/3	0/3	1/3	0/3
5000	1/3	0/3	2/3	1/3
10 000	1/3	1/3	2/3	2/3
50 000	3/3	2/3	3/3	2/3
Total	5/12	3/12	8/12	5/12

Abbreviations: BCSCs, breast cancer stem cells; miR, microRNA; NEK2, NIMA‐related kinase 2; NC, negative control.

### 3.11 miR‐128‐3p inhibits the Wnt signalling pathway by down‐regulating NEK2

**FIGURE 11:**
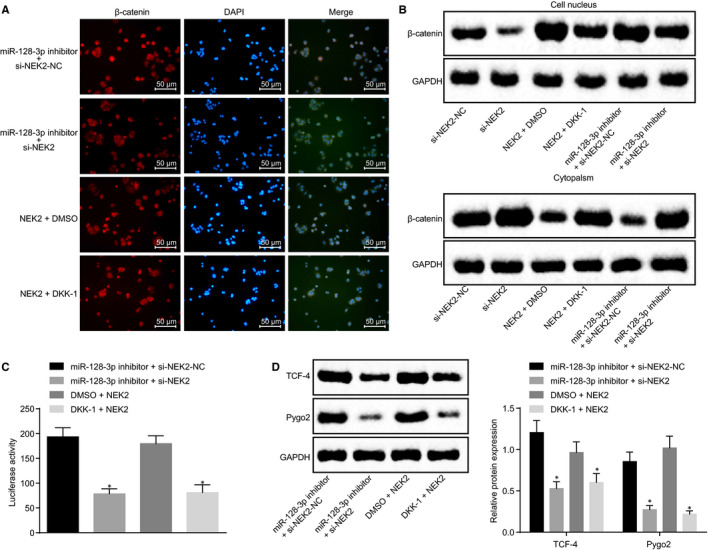
miR‐128‐3p inhibits the Wnt signalling pathway by down‐regulating NEK2. A, Detection of β‐catenin entering nuclear determined by immunofluorescence staining (×200); B, β‐catenin nuclear expression after transfection detected by Western blot analysis; C, Effect of transfected cells on TCF/LEF transcriptional activity; D, expression of downstream proteins of Wnt signalling pathway in different cells after transfection detected by Western blot analysis; *P < 0.05 compared with the corresponding NC group; the measurement data are expressed as mean ± standard deviation, and the data analysis among multiple groups was performed by one‐way analysis of variance. The experiment was repeated three times

The TOPFlash plasmid contains a firefly luciferase reporter gene, and the upstream promoter region of luciferase contains three repeat TCF‐binding sequences, which can regulate the expression of downstream luciferase according to the activity of β‐catenin. The TCF‐binding sequence in the TOPFlash plasmid is mutated, whereas other sequences are consistent with TOPFlash and are independent of β‐catenin activity. Therefore, TOP/TOPFlash is usually used as an index for activation of Wnt/β‐catenin signalling pathway in cells. The key point of Wnt/β‐catenin signalling pathway activation is that β‐catenin accumulation enters the nucleus and regulates the expression of genes combined with the transcription factor TCF/lethal factor (LEF). The nuclear translocation of β‐catenin was detected by immunofluorescence staining, finding that in BCSCs, in the DMSO + NEK2 group, the number of β‐catenin entering nuclear and TOPFlash activity have both increased; whereas they have both decreased in the DKK‐1 + NEK2 group, and the activation of the Wnt signalling pathway was inhibited. The miR‐128‐3p inhibitor + si‐NEK2‐NC group exhibited elevated β‐catenin entering the nucleus and the activity of TOPFlash. However, in the miR‐128‐3p inhibitor + si‐NEK2 group, the number of β‐catenin entering the nucleus and the activity of TOPFlash have both reduced, suggesting that silencing NEK2 can inhibit the activation of Wnt signalling pathway (Figure 11A‐C). At the same time, RT‐qPCR and Western blot analyses were carried out to detect the expression of the Wnt signalling pathway and downstream proteins. The results showed that compared with those in the DMSO + NEK2 group, the levels of TCF‐4 and Pygo2 were decreased in the DKK‐1 + NEK2 group, and Wnt signalling pathway activity was inhibited; compared with the miR‐128‐3p inhibitor + si‐NEK2‐NC group, the levels of TCF‐4 and Pygo2 were decreased and the activity of the Wnt signalling pathway was decreased in the miR‐128‐3p inhibitor + si‐NEK2 group (Figure 11D). All the results suggest that miR‐128‐3p can inhibit the Wnt signalling pathway activation via silencing of NEK2.

## 4 DISCUSSION

Breast cancer is the most common tumour among women worldwide, accompanied by high recurrence and mortality.^23^ Although many efforts and improvements have been made to promote the accuracy of diagnosis, patients still face poor quality of life and secondary lesions because of recurrence and metastasis of tumours.^24^, ^25^ Thus, in order to provide evidence for new therapeutic targets of breast cancer, the present study aims to clarify the roles of miR‐128‐3p and NEK2 in breast cancer, which reveals that miR‐128‐3 can inhibit the stem‐like cell characteristics including proliferation, migration, invasion and self‐renewal of BCSCs by down‐regulating NEK2.

Initially, this study found that miR‐128‐3p was down‐regulated in breast cancer tissues and cells and BCSCs. A previous study found that miR‐128‐3p was always poorly expressed in many kinds of cancers including hepatocellular carcinoma.^26^ In line with our study, another study showed that miR‐128‐3p was down‐regulated in both cells and tissues of oesophageal squamous cell carcinoma.^27^ Using bioinformatics tools in our study, we were able to find that NEK2 is highly expressed in breast cancer. Evidence shows that mitotic regulators including NEK2 are often up‐regulated in cancers, and that up‐regulated NEK2 is correlated with poor survival rate of patients suffering from breast cancer.^28^ Besides, by analysing the patient data of clinical breast cancer, a previous study confirmed that short survivals of breast cancer are closely related to the high expression of NEK2.^29^ Furthermore, based on these findings and the results of database prediction in our experiments, NEK2 was found to be a target gene of miR‐128‐3p. In lung cancer, NEK2 can be targeted and inhibited by miR‐128.^30^ Besides, a study conducted by Takahashi et al^31^ confirmed that expression of miR‐128 was negatively correlated with that of NEK2 expression in patients suffering from colorectal cancer. Based on these findings and the results of dual‐luciferase assay, the present study proved that NEK2 could be negatively regulated by miR‐128‐3p.

In addition, our findings suggest that overexpression of miR‐128‐3p can inhibit proliferation, migration and invasion of BCSCs through suppression of the Wnt signalling pathway by down‐regulation of NEK2, corresponding to decreased β‐catenin, CD24, CD44, Nanog and Oct‐4. A previous study has shown that through inactivation of the Wnt signalling pathway, Ganoderma lucidum is able to inhibit migration and proliferation of breast cancer cells.^32^ In line with our study, it has been proved that increased expression of NEK2 can activate the Wnt signalling pathway.^22^ A previous study suggests that the β‐catenin/Wnt signalling pathway which is closely related to stem cells also play a vital role in tumours.^33^ In addition, it has been proven that the expression level of β‐catenin in cancer stem cells can be down‐regulated by miR‐128.^34^ Then, another study indicates that CD24 and CD44 are cancer stem cells which can promote the development of breast cancer.^35^ Furthermore, it has been proven that quercetin 3‐methyl ether can down‐regulate the expression of Nanog, which is a gene associated with BCSCs.^36^ Oct‐4 and Nanog play vital roles in breast cancer, as decreased expression of Oct‐4 and Nanog can inhibit migration of BCSCs.^37^ A previous study has confirmed that when miR‐128‐3p is overexpressed, migration of invasive breast cancer cells can be inhibited.^38^ In addition, NEK2, which could alter cell migration, has been proven to be one of the most predictive genes that are related to metastasis‐free survival in breast cancer.^39^ Accumulating evidence suggests that decreased expression of NEK2 can repress amplification and proliferation of BCSCs.^40^, ^41^ In our study, when miR‐128‐3p was overexpressed or NEK2 was down‐regulated, the Wnt signalling pathway could be inactivated, thus suppressing cell migration, proliferation, and self‐renewal of BCSCs.

**FIGURE 12:**
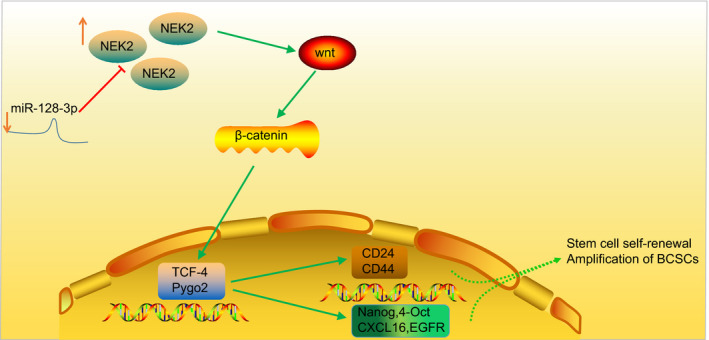
Regulation mechanism of miR‐128‐3p in BCSCs. miR‐128‐3p was significantly down‐regulated, whereas NEK2 was significantly up‐regulated in breast cancer cells and BCSCs. miR‐128‐3p could directly target and inhibit the expression of NEK2. Down‐regulated miR‐128‐3p activates the Wnt/β‐catenin signalling pathway by up‐regulating NEK2, thus enhancing the BCSC self‐renewal and amplification, as reflected by elevated expression of surface markers (CD24 and CD44), as well as stemness transcription factors (Nanog and﻿ Oct‐4)

Based on the previous researches, the present study confirmed that overexpressed miR‐128‐3p inhibited the proliferation, migration and invasion of BCSCs by suppressing the Wnt signalling pathway by down‐regulating NEK2 (Figure 12). Collectively, this study defines the potential role of miR‐128‐3p and NEK2 as a therapeutic target in breast cancer treatment by regulating the Wnt signalling pathway. However, the research is still at the preclinical stage, and future studies on the mechanism of action are required.

## CONFLICT OF INTEREST

The authors declare that they have no competing interests.

## AUTHOR CONTRIBUTIONS

Yuanwen Chen designed the study. Nian Wu collated the data and designed and developed the database. Lei Liu and Huaying Dong carried out data analyses and produced the initial draft of the manuscript. Yuanwen Chen and Xinao Liu contributed to drafting the manuscript. All authors have read and approved the final submitted manuscript.

## Data Availability

The data sets generated/analysed during the current study are available.
